# Management of hyperkalemia: strategic clinical actions in real-world practice

**DOI:** 10.1007/s10157-025-02728-2

**Published:** 2025-07-24

**Authors:** Takuya Fujimaru, Kazuhito Hirose, Masahiko Yazawa, Masahiko Nagahama, Csaba P. Kovesdy, Electrolyte Winter Seminar Collaborative Group, Takuya Fujimaru, Kazuhito Hirose, Masahiko Yazawa, Masahiko Nagahama, Yoshiro Fujita, Kyogo Kawada, Toshiaki Monkawa, Masatomo Ogata, Akihiro Ryuge, Yugo Shibagaki, Hideaki Shimizu, Hirofumi Sumi, Maho Terashita, Naoto Tominaga

**Affiliations:** 1 Department of Nephrology, St. Luke’s International Hospital, 9-1 Akashi-cho, Chuo-ku, Tokyo, 104-8560 Japan; 2 Department of General Medicine, Tsukuba Medical Center Hospital, Tsukuba, Japan; 3 Department of Internal Medicine, Yokohama General Hospital, Yokohama, Japan; 4 Division of Nephrology and Hypertension, Department of Internal Medicine, St. Marianna University School of Medicine, Kawasaki, Japan; 5 Division of Nephrology, Department of Medicine, University of Tennessee Health Science Center, Memphis, TN USA; 6 Memphis Veterans Affairs Medical Center, Nephrology Section, Memphis, TN USA

**Keywords:** Hyperkalemia, Potassium, Dyskalemia, Chronic kidney disease

## Abstract

This article is part of a review series on water and electrolyte disorders, based on the annual “Electrolyte Winter Seminar” for early-career nephrologists in Japan. The seminar features interactive case-based discussions, some of which are included as self-assessment questions. The fifth installment addresses the management of hyperkalemia. Hyperkalemia frequently occurs in patients with chronic kidney disease (CKD) and can become life-threatening when severe, necessitating prompt treatment regardless of its underlying cause. Renin–angiotensin system inhibitors (RASi) are a recognized risk factor for hyperkalemia in CKD; however, discontinuing RASi in response to elevated potassium levels may adversely affect patient outcomes. Although there are no formal criteria distinguishing acute from chronic hyperkalemia, symptoms presentation and potassium levels offer a practical guide for clinical management. This review covers standard treatment strategies for severe (symptomatic or acute) hyperkalemia in emergency and inpatient settings and discusses how to manage mild-to-moderate (asymptomatic or chronic) cases in CKD patients while continuing RASi therapy.

## Introduction

The management of severe electrolyte disturbances such as hyperkalemia should be guided by clinical urgency rather than rigid diagnostic algorithms. Prompt recognition and treatment are critical for preventing serious outcomes like arrhythmias. To avoid delays, clinical criteria for severe hyperkalemia must be established clearly—considering serum potassium and electrocardiographic findings and neuromuscular symptoms. However, most cases encountered in clinical practice are not immediately life-threatening. Many involve chronic hyperkalemia in patients with chronic kidney disease (CKD), where potassium levels may fluctuate but stay near the normal range. In such cases, the primary goal is to prevent recurrence rather than rapidly lower potassium. Although distinguishing between acute and chronic hyperkalemia is clinically relevant, no universal definitions exist; clinicians typically rely on symptoms and potassium levels to guide decisions. Given the reno- and cardio-protective benefits of renin–angiotensin system inhibitors (RASi), maintaining their use through appropriate potassium control is vital in CKD management. If temporary discontinuation is necessary, early resumption should be prioritized. This review presents a practical, updated approach to hyperkalemia management, emphasizing individualized care.

## Management of hyperkalemia

### Classification of the chronicity and severity of hyperkalemia

Hyperkalemia is generally classified as acute or chronic based on clinical context and underlying mechanisms. However, no standardized criteria define chronicity concerning potassium levels magnitude, duration, or frequency. In practice, the presence or absence of symptoms often serves as a pragmatic indicator.

Here, acute hyperkalemia refers to a symptomatic, potentially life-threatening condition requiring immediate intervention. It typically results from a rapid, sustained rise in potassium that overwhelms homeostasis. Common causes include acute kidney injury (AKI), rhabdomyolysis, or additional insults in CKD that impair potassium excretion. In contrast, chronic hyperkalemia is usually asymptomatic and detected incidentally during routine labs tests, especially in patients with CKD, diabetes, or heart failure. While potassium levels may rise mildly in CKD, they do not necessarily remain persistently elevated [1–3]. Chronic cases typically involve mild-to-moderate, recurrent, or sustained elevations but can acutely deteriorate, resulting in symptomatic and severe hyperkalemia.

**Fig. 1:**
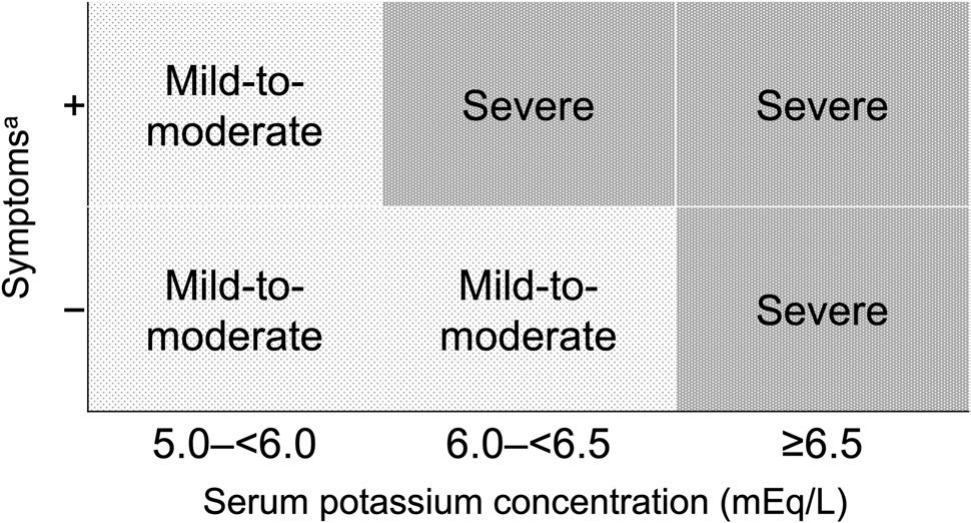
Hyperkalemia severity: expert opinion-based risk classification. **a**. Electrocardiogram changes or muscle weakness

Although higher potassium levels increase the likelihood of electrocardiographic (ECG) abnormalities [4], this relationship is influenced by calcium, magnesium, acid–base status, etc. Some patients show no ECG changes even at potassium levels of 8.5 mEq/L, highlighting the limited sensitivity of ECG for detecting hyperkalemia [5, 6]. However, ECG abnormalities, muscle weakness, or paralysis are reliable markers of symptomatic and severe hyperkalemia [7]. The Kidney Disease: Improving Global Outcomes (KDIGO) consensus classifies hyperkalemia as mild, moderate, or severe, based on serum potassium and ECG changes [8]. While no formal criteria distinguish acute from chronic hyperkalemia, the KDIGO framework offers a practical clinical guide. Herein, hyperkalemia is defined as serum potassium ≥ 5.0 mEq/L. We propose classifying cases as mild-to-moderate or severe, based on potassium levels and the presence or absence of ECG abnormalities or neuromuscular symptoms (Fig. 1). Regarding chronicity, acute hyperkalemia typically presents as symptomatic and severe, whereas chronic hyperkalemia is often asymptomatic with mild-to-moderate hyperkalemia severity. Although not definitive, this model offers a clinically useful basis for assessment and management.

### Essential clinical information on hyperkalemia

Table 1 outlines key clinical information for managing hyperkalemia. Although treatment should not be delayed, pseudohyperkalemia—such as from hemolysis—should be excluded. Whenever feasible, proper blood sampling techniques should be used, and repeat potassium testing with arterial blood gas analysis may be necessary. Vital signs should be checked for abnormalities such as bradycardia or hypotension. Muscular symptoms, including weakness or paralysis, may indicate severe hyperkalemia [9]. Peaked T-waves, observed in lead V2 on ECG, are common [10], and bradycardia and QRS prolongation are critical warning signs. Hyperglycemia, metabolic acidosis (high-anion gap or hyperchloremic), and urine output must be evaluated for treatment efficacy.

**Table 1:** Key clinical information on hyperkalemia

Pseudohyperkalemia? (Signs of hemolysis?)	LDH, AST, checking other conditions (CBC, fist clenching, storage conditions including temperatures and time)
Stable vital signs?	BP, HR, respiratory rate, SpO_2_
Muscle symptoms?	Muscle weakness and paralysis (especially in proximal lower limb muscles)
Blood glucose level? (Hyperglycemia?)	Blood glucose level
Acid–base disturbance? (Metabolic acidosis?)	Blood gas (arterial blood gas; if possible) pH, pCO_2_, HCO_3_^−^, anion gap (Na^+^ and Cl^−^)
Urine output?	AKI due to pre- and post-renal cause, oliguric or anuric/ESKD on dialysis
ECG changes?	Peaked T-wave, bradycardia, prolonged PR interval, prolonged QRS duration, flattened P wave

*AKI* acute kidney injury, *AST* aspartate aminotransferase, *BP* blood pressure, *CBC* complete blood count, *Cl*^*−*^ concentration of chloride level, ECG electrocardiogram, *ESKD* end-stage kidney disease, *Na*^+^ concentration of sodium level, *HR* heart rate, *HCO*_*3*_^*−*^ concentration of bicarbonate level, LDH lactate dehydrogenase, *pCO*_*2*_ partial pressure of carbon dioxide, *pH* potential of hydrogen, *SpO*_*2*_ peripheral capillary oxygen saturation

### Caution before treating hyperkalemia

When hyperkalemia reflects an actual increase in total body potassium, treatment should promote its excretion. However, in rare cases like hyperkalemic periodic paralysis, hyperkalemia results from a shift from intracellular to extracellular compartments without a net potassium gain. In such cases, aggressive removal may cause severe hypokalemia once the shift resolves. Similarly, in pseudohyperkalemia, serum potassium is normal in vivo, so potassium-lowering therapy may lead to iatrogenic hypokalemia.

## Severe hyperkalemia

**Fig. 2:**
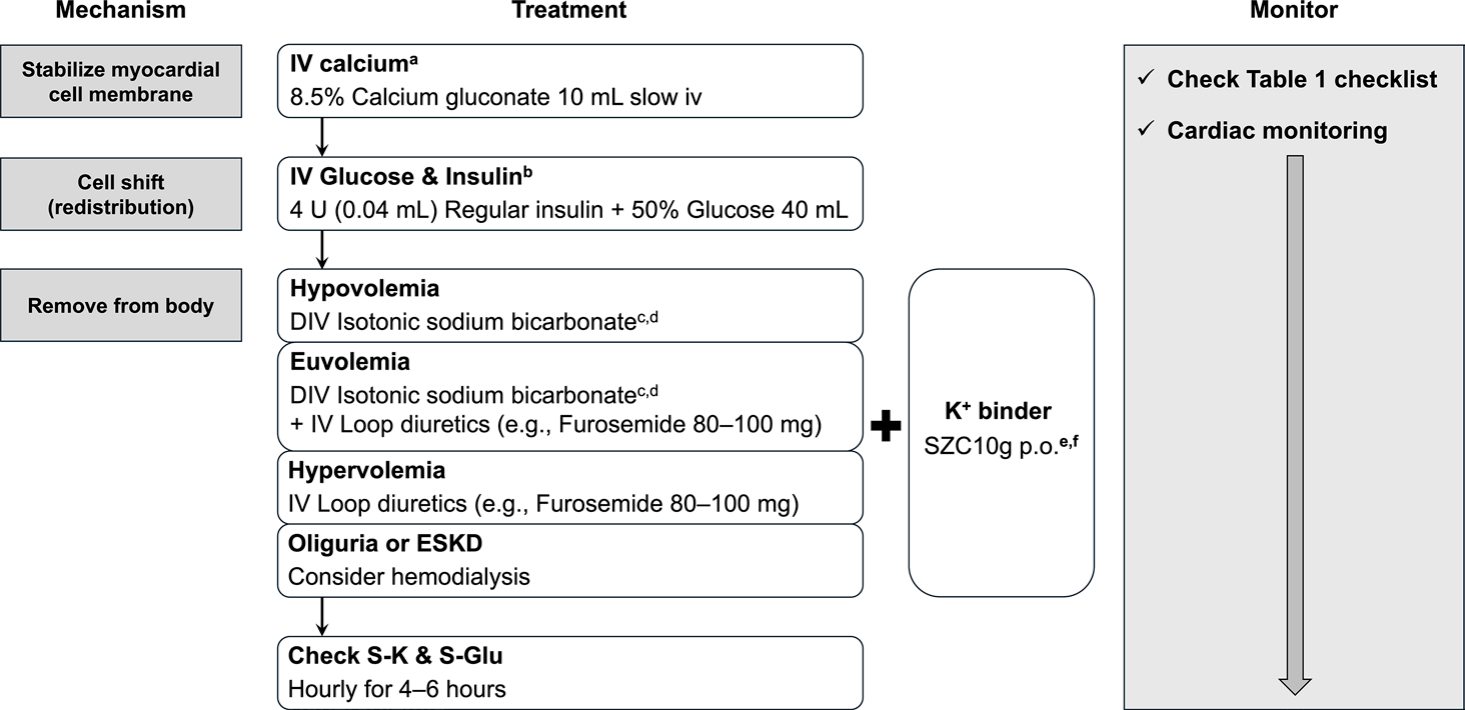
Management of severe hyperkalemia. **a** Consider repeating the doses if electrocardiogram changes persist after 5 min or recur. **b** Administer every 2–4 h, as necessary. **c** If unavailable, balanced fluid are preferred over normal saline. **d** In cases of DKA or HHS, opt for normal saline or balanced fluids instead of isotonic bicarbonate. **e** Continue 10 g three times daily for 48 h, if required (in non-hemodialysis patients). **f** Initial dose is 5 g in hemodialysis patients. *DIV* intravenous drip, *DKA* diabetic ketoacidosis, *ESKD* end-stage kidney disease, *HHS* hyperosmolar hyperglycemic state, *IV* intravenous, *p.o.* per os, *S-Glu* serum glucose concentration, *S-K* serum potassium concentration, *SZC* sodium zirconium cyclosilicate

This is an acute, symptomatic condition with elevated serum potassium and either ECG abnormalities or muscle weakness. As this definition is based on clinical presentation rather than etiology, the management strategies are broadly applicable regardless of the underlying cause. Figure 2 outlines the treatment approach.

### Intravenous calcium

Intravenous calcium is the first-line treatment for severe hyperkalemia, even when serum calcium is normal. The muscle weakness and cardiac effects stem from neuromuscular transmission. Membrane excitability, governed by resting potential and sodium channel activation, is influenced by extracellular potassium. Elevated potassium reduces the intracellular–extracellular gradient, making the resting potential less negative and partially depolarizing the cell. This initially increases excitability but eventually inactivates sodium channels, impairing cardiac conduction and causing neuromuscular weakness or paralysis. Calcium raises the threshold potential without altering the resting potential. By restoring the narrowed gap between resting and threshold potentials, calcium reduces excitability and stabilizes cardiac function [11].

International guidelines recommend 10 mL of 10% calcium gluconate. In Japan, where only 8.5% formulation is available, 10 mL of 8.5% is used. Infusion over 2–3 min should be done with continuous cardiac monitoring. Effect begins within 1–3 and lasts 30–60 min [12]. If ECG changes persist or recur after 5 min, repeat dosing should be considered [8]. In patients on digitalis, calcium must be used cautiously due to the risk of enhanced cardiac toxicity [1]. It should not be mixed with bicarbonate-containing solutions to avoid calcium carbonate precipitation.

### Intravenous glucose and insulin (glucose–insulin therapy)

Insulin lowers plasma potassium by promoting cellular uptake, particularly in skeletal muscle and liver. Glucose is co-administered to prevent hypoglycemia; however, insulin alone may suffice in hyperglycemic patients. The traditional regimen includes 10 units of regular insulin with 50 mL of 50% glucose [13]. Recent studies show that 5 units of regular insulin with the same glucose volume is equally effective [14, 15] and reduces hypoglycemia risk [16]. Accordingly, the KDIGO recommends 5 units of regular insulin with 50 mL of 50% glucose (1 unit per 5 g of glucose) [8]. In Japan, where 50% glucose is only available in 20-mL vials, 4 units of regular insulin with 40 mL of 50% glucose is commonly used. In some hospitals, syringes are marked in 0.02-mL increments, making it difficult to measure odd-numbered unit doses accurately. Blood glucose should be monitored hourly for 4–6 h after glucose–insulin therapy due to hypoglycemia risk [17, 18], which is more common in patients with kidney impairment, no diabetes, low baseline glucose, low body weight, or female sex [15]. Regular insulin is typically supplied at 100 units/mL. Standardized preparation of glucose–insulin therapy is essential to prevent errors, such as hypoglycemia from incorrect dosing. Hospitals should implement standard protocols. Since hemodialysis setup may take 1–2 h, glucose–insulin therapy might need to be repeated every 2–4 h. Repeated dosing increases the risk of hypoglycemia [19], requiring careful glucose monitoring. For continuous infusion, 10% glucose (500 mL) with 10 units of regular insulin is used, with the infusion rate adjusted based on the total infusion volume, hypoglycemia risk, and clinical context. Short-acting synthetic insulins (lispro, aspart, glulisine) are less affected by renal function, as they are degraded enzymatically rather than renally. Some experts recommend them over regular insulin to reduce hypoglycemia risk [20]. In Japan, only aspart is approved for intravenous use.

### Isotonic sodium bicarbonate

In anuric dialysis patients, sodium bicarbonate has limited efficacy in acutely lowering serum potassium, even with metabolic acidosis [21, 22]. In contrast, in non-anuric patients, it may enhance potassium excretion in the distal nephron by increasing sodium delivery, urinary flow, and lumen electronegativity. It should be used in hypovolemic patients and, even in euvolemia, augment the renal potassium-lowering effects of diuretics and help prevent volume depletion. In Japan, a 1.26% sodium bicarbonate—containing 150 mEq/L of sodium and bicarbonate—is available as an isotonic solution. If unavailable, a 7.0% or 8.4% sodium bicarbonate solution can be diluted with glucose or sterile water. For example, mixing five 20-mL ampoules of 7.0% sodium bicarbonate (Meylon®) with 450 mL of 5% glucose yields a solution with 151 mEq/L of sodium and bicarbonate.

### Loop diuretic

Loop diuretics increase distal sodium delivery to the collecting duct, enhancing potassium excretion in patients with normal or mildly to moderately impaired kidney function. However, renal impairment is often unpredictable with severe hyperkalemia. Therefore, we recommend administering the maximum effective “ceiling” dose (e.g., 80–100 mg of furosemide), similar to a furosemide challenge test [23, 24]. Because most AKI cases exhibit diuretic resistance, combining loop diuretics with isotonic sodium bicarbonate is preferred, as this increases urine flow and enhances luminal electronegativity. Hydration is unnecessary in patients with fluid overload.

### Sodium zirconium cyclosilicate (SZC)

SZC is a highly selective cation exchanger that binds potassium in the intestines in exchange for sodium and hydrogen [25]. Although approved only for chronic hyperkalemia, limited data suggest it can acutely lower serum potassium in severe cases [26–28]. In a Japanese randomized trial of non-dialysis patients with potassium levels between 5.1 and 6.5 mEq/L, 10 g of SZC reduced potassium by 0.37 mEq/L after 1 h [29]. Although not approved as monotherapy for severe hyperkalemia, SZC can be used adjunctively, including in patients with end-stage kidney disease (ESKD). Under insurance coverage, non-hemodialysis patients can receive 10 g up to three times daily for the first 2 days. For those with impaired consciousness or difficulty taking oral medications, SZC can be administered via nasogastric tube. Precautions include the risk of iatrogenic hypokalemia due to its potency and potential gastrointestinal symptoms.

### Hemodialysis

For severe hyperkalemia, intermittent hemodialysis should be considered first-line kidney replacement therapy, as it removes potassium more effectively than continuous kidney replacement therapy (CKRT) [30]. A dialysis session using dialysate with a potassium concentration of 1 mEq/L removes 70–100 mEq of potassium via diffusion and convection [31, 32]. The greatest reduction (1.2–1.5 mEq/L) occurs in the first hour, when the serum–dialysate gradient is highest [31, 32]. Potassium typically reaches its nadir after ~ 3 h, with ~ 1 mEq/L drop in the first hour, followed by another 1 mEq/L decrease over the next 2 h. The decline slows as the gradient narrows. In the absence of ongoing potassium release (e.g., tumor lysis syndrome or rhabdomyolysis), 3–4 h of hemodialysis is generally effective. Post-dialysis rebound may occur due to intracellular shift, requiring close monitoring. CKRT may be preferred in patients with hemodynamic instability or sustained potassium release from tissue injury (e.g., rhabdomyolysis, tumor lysis syndrome, hemolysis) [30]. Dialysis setup requires time; depending on the facility, 1–2 h may be needed to secure vascular access, staff, and equipment.

## -Self-assessment question 1-

A 75-year-old man with diabetes and hypertension was diagnosed with dermatomyositis 1 month ago and started on prednisolone 20 mg/day. Prior to steroid, his HbA1c was 6.4%, serum creatinine was 1.9 mg/dL, estimated glomerular filtration rate (eGFR) was 27 mL/min/1.73 m^2^, and potassium fluctuated around 5.5 mEq/L. Medications included telmisartan 80 mg/day, hydrochlorothiazide 12.5 mg/day, esaxerenone 2.5 mg/day, and azelnidipine 16 mg/day.

He presented with anorexia and fatigue. Vital signs were stable except for mild tachycardia. Physical exam showed decreased skin turgor, dry axillae, and dry oral mucosa, without muscle symptoms. Labs showed blood urea nitrogen (BUN) 85.3 mg/dL, creatinine 3.4 mg/dL, pH 7.299, pCO₂ 44.2 mmHg, bicarbonate 21.1 mEq/L, sodium 129 mEq/L, potassium 6.6 mEq/L, chloride 97 mEq/L, glucose 853 mg/dL, and calcium 2.8 mEq/L. Urinary ketones were negative, and bedside 3-hydroxybutyric acid was 0.3 mmol/L, indicating no elevation. Hyperosmolar hyperglycemic state (HHS) was diagnosed. ECG was unremarkable.

## Question: which treatment is most appropriate for managing hyperkalemia?

A. 40 mL of 50% glucose solution and 4 units of regular insulin.

B. Administering 10 mL of 8.5% calcium gluconate.

C. Administering of 4 units of regular insulin and initiating buffered crystalloid hydration.

D. Initiating SZC (10 g) three times a day.

The correct answer is C.

Due to hyperglycemia, additional glucose is unnecessary (A). Calcium gluconate is inappropriate given the hypercalcemia (B). In diabetic ketoacidosis (DKA) or HHS, potassium deficits of 300–600 mEq arise from osmotic diuresis, yet serum potassium may appear elevated due to insulin deficiency and hyperosmolarity, which shift potassium extracellularly [33]. Insulin promotes intracellular potassium shift and lowers serum potassium, so potassium-lowering agents like SZC may cause hypokalemia (D).

Consensus guidelines from the American Diabetes Association, Joint British Diabetes Societies, American College of Clinical Endocrinology, and Diabetes Technology Society recommend initial HHS treatment with isotonic fluids (e.g., buffered crystalloids) to restore volume and stabilize hemodynamics, followed by potassium evaluation and low-dose intravenous insulin if potassium ≥ 3.5 mEq/L [34]. Therefore, 4 units of regular insulin with buffered crystalloid hydration (C) is appropriate. While insulin also help to correct acidosis, routine bicarbonate use is not advised due to the risk of hypokalemia [34].

## Mild-to-moderate hyperkalemia

The kidneys play a central role in potassium homeostasis, and hyperkalemia risk increases as kidney function declines [35–38]. Thus, many patients with mild-to-moderate hyperkalemia have CKD. Although the distinction between acute and chronic hyperkalemia in not well defined, asymptomatic, mild-to-moderate elevations—common in CKD—are generally considered chronic. This section addresses the management of such cases, focusing not on rapid correction but on preventing recurrence.

### Importance of avoiding dose reduction or discontinuation of RASi

RASi offer cardiovascular and renoprotective benefits in CKD patients [39–41], but hyperkalemia is a common adverse effect [40, 42]. In practice, 15–30% of patients discontinue RASi when eGFR falls below 30 mL/min/1.73 m^2^, often due to hyperkalemia or AKI [43]. However, stopping RASi significantly increases all-cause mortality and cardiovascular risk [40, 43, 44]. Managing other hyperkalemia risk factors is, therefore, essential to avoid unnecessary discontinuation. Table 2 outlines key interventions to consider before reducing or stopping RASi in patients without severe hyperkalemia.

**Table 2:** Steps to take before reducing or discontinuing RASi in mild-to-moderate hyperkalemia

**Lifestyle**
Review K^+^-elevating medications (except RASi)
Assess and restrict dietary K^+^ levels if needed
Evaluate and manage constipation
**CKD management other than hyperkalemia**
Volume overload; use of diuretics/SGLT2i
Volume depletion/hypotension; reduce or stop diuretics, SGLT2i, or antihypertensives
Correction of metabolic acidosis
Insulin deficiency: blood glucose control
**Direct K**^**+**^** lowering agents**
K^+^ binder: SPS, CPS, patiromer, SZC

*CKD* chronic kidney disease, *CPS* calcium polystyrene sulfonate, *K*^+^ potassium, *RASi* renin–angiotensin system inhibitor, *SGLT2i* sodium–glucose cotransporter 2 inhibitor, *SPS* sodium polystyrene sulfonate, *SZC* sodium zirconium cyclosilicate

Discontinuation threshold varies by specialty. Nephrology guidelines, including KDIGO and the Japanese Society of Nephrology (JSN), define hyperkalemia as a serum potassium > 5.5 mEq/L and may consider RASi reduction or discontinuation in CKD patients at this level [45]. In contrast, cardiology guidelines, such as those from the European Society of Cardiology, recommend continuation up to 6.5 mEq/L, using potassium binders as first-line, and discontinuing only above this threshold [46].

Although hyperkalemia often leads to temporary discontinuation of RASi, recent evidence suggests that the risk of hyperkalemia recurrence after reinitiation is not significantly increased with appropriate monitoring [47].

### Review of potassium-elevating medications (excluding RASi)

A thorough review of the patient’s medication history is essential for hyperkalemia management. Table 3 lists potassium-elevating drugs. In Japan, non-steroidal anti-inflammatory drugs (NSAIDs) are now available over-the-counter (OTC), so prescribed and OTC medications should be reviewed. β-Blockers may contribute to hyperkalemia via impaired intracellular potassium shift. Reduced aldosterone activity—critical for potassium excretion in principal cells of the collecting ducts—can also cause hyperkalemia, as several drugs suppress aldosterone secretion or blunt its action. While many are essential for CKD or cardiovascular disease, management typically involves avoiding NSAIDs or adjusting antibiotic regimens.

**Table 3:** Medications that cause hyperkalemia

**1. Abnormal potassium distribution**
Beta-blockers and hyperosmolar substances (e.g., immunoglobulins, radiocontrast agents) in ESKD/AKI
**2. Decreased kidney excretion due to hypoaldosteronism**
**2-1.Reduced aldosterone secretion:** ACEi, ARB, direct renin inhibitors, aldosterone synthase inhibitors, NSAIDs, heparin
**2-2.Decreased responsiveness to aldosterone:** MRA, trimethoprim, pentamidine, and CNI
**3 Others**
Penicillin G (one million units of penicillin G contains 1.53 mEq of potassium) [93]

*ACEi* angiotensin-converting enzyme inhibitor, *AKI* acute kidney injury,* ARB* angiotensin II receptor blockers, *CNI* calcineurin inhibitors, *ESKD* end-stage kidney disease, *MRA* mineralocorticoid receptor antagonist, *NSAIDs* non-steroidal anti-inflammatory drugs

### Assess and restrict dietary potassium if necessary

Dried fruits, seaweed, nuts, and avocados are high in potassium [48]. Soaking frozen foods or boiling them can reduce potassium content by about half [49, 50]. However, studies show only a weak correlation between dietary potassium and serum levels in CKD patients [51, 52]. In fact, increased fruit and vegetable intake may improve blood pressure, reduce acidosis, and slow CKD progression without raising serum potassium [53]. A Japanese cohort study—including patients without CKD, with non-dialysis CKD, and on hemodialysis—found no association between fruit and vegetable intake and serum potassium. Compared to daily intake, occasional intake was linked to 25% higher mortality, and rare intake to 60% higher mortality, regardless of CKD stage [54]. The KDIGO guideline advises against restricting natural potassium-rich foods, which may be harmful in early CKD [45]. Some experts also emphasize potassium bioavailability [53]: potassium in plant-based foods is less absorbable than in processed foods. Highly processed food—such as those with potassium chloride or phosphate, meat, dairy, juices, and salt substitutes—contain more absorbable potassium. Salt substitutes made with potassium chloride, used to reduce sodium intake, may inadvertently increase potassium load, especially in CKD patients unaware of their content. Therefore, dietary education is essential, with emphasis on avoiding processed and uncooked foods rather than broadly limiting potassium-rich foods.

### Evaluate and manage constipation

In healthy adults, 90%–95% of ingested potassium is excreted in urine and 5%–10% in stool. In contrast, fecal potassium excretion in hemodialysis patients is about threefold higher and can reach up to 80% of intake (up to 3,000 mg/day) [55–57]. Constipation is more common in CKD patients, especially in advanced stages [58]. Notably, laxative use in the year before ESKD onset—regardless of type—has been independently linked to a lower incidence of hyperkalemia [59]. Thus, evaluating and managing constipation is essential in CKD-related hyperkalemia management.

### Volume overload: use of diuretics/sodium–glucose cotransporter 2 (SGLT2i)

Volume overload is common in moderate-to-advanced CKD and is associated with hypertension, heart failure, and edema. Diuretics are often prescribed to manage blood pressure and relieve volume overload [60]. Loop diuretics increase potassium excretion in patients with normal to moderately impaired kidney function, while thiazides are generally more effective in lowering serum potassium in those with relatively preserved renal function [61]. Notably, thiazides can reduce potassium even in advanced CKD [62, 63]. Therefore, a multi-targeted diuretic approach may help manage hyperkalemia and fluid overload.

Acetazolamide, a carbonic anhydrase inhibitor, increases distal sodium delivery and inhibits bicarbonate reabsorption in the proximal tubule, enhancing luminal electronegativity and urinary potassium excretion. This shifts the acid–base balance toward metabolic acidosis. Acetazolamide has gained renewed attention in heart failure due to its potassium-lowering-effect. In sub-analysis of the ADVOR trial, heart failure patients receiving acetazolamide and loop diuretics had a 0.2 mEq/L greater potassium reduction on day 3 versus placebo [64, 65].

Recent studies show that SGLT2i reduce hyperkalemia risk [66, 67] and improve volume status in CKD patients [68]. They may, thus, be useful in hyperkalemia management in this population. For details, see our related review [69].

### Volume depletion/hypotension: reduce or stop diuretics, SGLT2i, or antihypertensives

Volume depletion and hypotension lower GFR, increasing hyperkalemia risk. In such cases, consider reducing or stopping diuretics or SGLT2i. If hypotension occurs without volume depletion, adjusting or discontinuing antihypertensives may be needed. However, beta-blockers—essential for heart disease—should not be stopped without careful evaluation, unlike calcium channel or alpha-blockers [46]. If RASi are discontinued, they should be resumed once volume or blood pressure normalizes.

To reduce hyperkalemia risk in CKD, set lower thresholds for blood pressure and body weight, and instruct patients, families, or home care nurses on adjusting antihypertensives and diuretics. Patients should also be advised to stop SGLT2i and RASi during sick days [45].

### Correction of metabolic acidosis

Metabolic acidosis, especially hyperchloremic, promotes potassium efflux from cells [70]. Low serum bicarbonate is a recognized hyperkalemia risk factor in CKD [71], and correcting acidosis may help lower potassium [72, 73]. The JSN recommends treatment of metabolic acidosis when [HCO_3_^−^] falls below 22 mEq/L [74]. Blood gas analysis is required to measure [HCO_3_^−^], though total CO₂ may be used, albeit less commonly in Japan. The [Na⁺] − [Cl⁻] difference can help identify hyperchloremic metabolic acidosis [42, 75]. This can be illustrated by the serum anion gap equation:

Serum anion gap (mEq/L) = [Na⁺] (mEq/L) − [Cl⁻] (mEq/L) − [HCO₃⁻] (mEq/L), rearranged as.

[Na⁺] (mEq/L) − [Cl⁻] (mEq/L) = Serum anion gap (mEq/L) + [HCO₃⁻] (mEq/L).

In hyperchloremic metabolic acidosis, the serum anion gap remains normal, so [Na⁺] − [Cl⁻] decreases. In high-anion gap metabolic acidosis, [HCO₃⁻] falls but is offset by an increased anion gap, keeping [Na⁺] − [Cl⁻] stable. Thus, a low [Na⁺] − [Cl⁻] suggests hyperchloremic metabolic acidosis. Assuming a target [HCO₃⁻] of 22 mEq/L and a normal serum anion gap of 12 mEq/L, [Na⁺] − [Cl⁻] < 34 mEq/L may indicate low bicarbonate levels.

Because albumin is the major unmeasured anion, hypoalbuminemia lowers anion gap—by approximately 2.5 mEq/L for every 1 g/dL decrease below 4 g/dL. Therefore, normal [Na⁺] − [Cl⁻] is lower in hypoalbuminemia [42].

### Insulin deficiency: blood glucose control

Insulin promotes intracellular potassium uptake; thus, insulin deficiency can result in hyperkalemia. Hyperglycemia reflects insulin deficiency; thus, blood glucose control may help mitigate hyperkalemia.

### Direct potassium-lowering agents: sodium polystyrene sulfonate (SPS), calcium polystyrene sulfonate (CPS), patiromer, SZC

Polystyrene sulfonate (PS) is a cation-exchange resin used to treat hyperkalemia. There are two forms: sodium-based SPS and calcium-based CPS. Both are cross-linked polymers containing sulfonic acid groups that exchange bound cations for potassium in the colon, enhancing fecal excretion [76]. Although CPS is not approved in the United States, SPS and CPS similarly reduce serum potassium levels [77]. However, SPS significantly lowers serum calcium and increases serum sodium and atrial natriuretic peptide levels [76]. Thus, CPS may be more suitable for CKD patients with fluid overload or more impaired renal function. Notably, long-term randomized controlled trials for SPS are lacking. While they are inexpensive and widely used, they carry a rare but serious risk of intestinal necrosis [78, 79]. In Japan, concurrent use with sorbitol is generally avoided due to this risk.

Patiromer is another non-absorbed polymer that exchanges potassium for calcium in the colon [80–82]. Approved in the United States in 2015, it became available in Japan in 2025 following a phase III trial involving patients with serum potassium levels of 5.5–6.5 mEq/L [83].

As noted earlier, SZC is highly selective cation exchanger that binds potassium in exchange for sodium and hydrogen in the intestines [25, 84]. In vitro studies suggest a potassium-binding capacity up to nine times that of SPS [85]. The National Institute for Health and Care Excellence guidelines recommend SZC for managing persistent hyperkalemia in CKD [86]. Though costly, its use has been associated with reduced overall healthcare costs in the US by facilitating continued RASi therapy in CKD patients [87]. SZC is also effective in dialysis patients, as demonstrated in the DIALIZE trial, where administration on non-dialysis days maintained potassium control without increasing interdialytic weight gain [88]. Compared with other binders, SZC causes fewer gastrointestinal side effects. However, due to its radiopaque nature, it may interfere with imaging and body composition assessments [89, 90]. Dose-dependent edema is another concern, particularly with the 15 g daily dose recommended during the initial treatment (Lokelma® prescribing information, Wilmington, DE: AstraZeneca; May 2018).

Key differences among potassium binders are summarized in Table 4.

**Table 4:** **Comparisons of potassium binders** [94–98]

	SPS	CPS	Patiromer	SZC
Mechanism of action	Sodium-based cation-exchange resin Binds not only K^+^ but also other cations, such as NH_4_^+^, Ca^2+^, and Mg^2+^	Calcium-based cation-exchange resin	Calcium-based cation-exchange polymer Binds not only K^+^ but also Mg^2+^	Non-polymeric inorganic cation-exchange crystals Exchange K^+^ with H^+^ and Na^+^
Site of action	Colon	Colon	Colon	The entire intestines
Onset of the action	Hours to days	Hours to days	7 h	1 h
Timing of the medication	Separate by at least 3 h from other medications^a^	Separate by at least 3 h from other medications^a^	Separate by at least 3 h from other medications^a^	Separate by at least 2 h from other medications^a^
Adverse effects				
Gastrointestinal	Diarrhea [99], nausea, vomiting, constipation, stomach discomfort, loss of appetite, abdominal pain, intestinal perforation [100, 101], intestinal ulcer [102], and intestinal necrosis [103–105]	Constipation, nausea, loss of appetite, and stomach discomfort, intestinal perforation, intestinal obstruction [106, 107], colon ulcer [106]	Abdominal distress, constipation, diarrhea, flatulence, nausea, intestinal perforation, and intestinal obstruction	Constipation
Acid–base and electrolyte	Alkalosis [108], hypernatremia [109], hypocalcemia [108], hypomagnesemia	Hypercalcemia [110], hypomagnesemia	Hypomagnesemia, hypophosphatemia	No significant adverse effects
Others	Edema, increased blood pressure, and heart failure	No significant adverse effects	No significant adverse effects	Congestive heart failure and edema

a. Not included in the Japanese package insert

*CPS* calcium polystyrene sulfonate, *SPS* sodium polystyrene sulfonate, *SZC* sodium zirconium cyclosilicate

### Reduction in dose or discontinuation of RASi

If serum potassium remains elevated despite the interventions in Table 2, reducing the dose or discontinuing RASi may be necessary. If hyperkalemia improves, prompt reinitiation is essential. One study found that early RASi reinitiation reduced the risk of mortality, cardiovascular events, and ESKD without increasing the risk of readmission due to severe hyperkalemia or AKI [91]. Therefore, reinitiating RASi should be considered as soon as patients are eligible.

## -Self-assessment question 2-

An 85-year-old man with a history of right nephrectomy for renal cell carcinoma at age 70 and longstanding hypertension is followed for CKD (serum creatinine 2.0 mg/dL, eGFR 25 mL/min/1.73 m^2^). His medications include valsartan 160 mg/day, amlodipine 10 mg/day, carvedilol 10 mg/day, trichlormethiazide 2 mg/day, esaxerenone 2.5 mg/day, and sodium bicarbonate 0.5 g/day. During an annual health checkup, labs showed: BUN 80 mg/dL, serum creatinine 2.27 mg/dL, sodium 137 mEq/L, potassium 5.9 mEq/L, chloride 106 mEq/L, and glucose 104 mg/dL. He had been fasting since the previous day and was referred to nephrology the same day. On examination, blood pressure 118/52 mmHg, heart rate 71 beats/min, ECG was normal, and there were no muscle symptoms or leg edema.

## Question: which of the following is the most appropriate outpatient treatment?

A. Discontinue valsartan.

B. Encourage him to start eating and drinking water, and follow-up with a blood test in a week.

C. Prescribe an SGLT2i.

D. Prescribe furosemide.

The correct answer is B.

Fasting reduces intravascular volume [92] and suppresses insulin, impairing potassium excretion by lowering GFR, distal sodium delivery, and urinary flow. Herein, fasting-induced volume depletion and reduced renal function caused hyperkalemia. Prescribing furosemide (D) or initiating an SGLT2i (C) would worsen volume status. As this is not a sick day, oral intake may restore volume and correct potassium levels. Discontinuing valsartan (A) would be premature. The appropriate approach is to resume eating and drinking, with follow-up labs in 1 week (B). At follow-up, BUN was 63.2 mg/dL, creatinine was 2.0 mg/dL, sodium was 138 mEq/L, potassium was 5.0 mEq/L, and chloride was 108 mEq/L.

## Conclusion

Treatment and diagnostic algorithms for hyperkalemia should be addressed separately. For severe hyperkalemia, promptly administer intravenous calcium gluconate, followed by glucose and insulin. Subsequently, based on volume status, consider sodium bicarbonate or diuretics. If feasible, administer SZC orally. If hyperkalemia persists, initiate hemodialysis. For non-severe hyperkalemia in CKD, control potassium without reducing or discontinuing RASi. If RASi must be withheld, resume as soon as hyperkalemia resolves.

## Data Availability

Not applicable.
